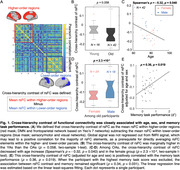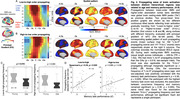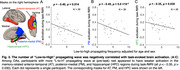# Resting‐state activity across cortical hierarchies provides readiness for memorizing in aging humans

**DOI:** 10.1002/alz.088685

**Published:** 2025-01-09

**Authors:** Feng Han, Xi Chen, Leah Varghese, Kaitlin Cassady, Trevor Chadwick, Suzanne Mason, William J. Jagust

**Affiliations:** ^1^ University of California, Berkeley, Berkeley, CA USA; ^2^ Lawrence Berkeley National Laboratory, Berkeley, CA USA

## Abstract

**Background:**

The neural basis of memory aging remains elusive. The default mode network (DMN) supports memory encoding and retrieval, and its connectivity decreases in aging. Young adults with larger differences in resting‐state functional connectivity (rsFC) between higher‐order DMN and lower‐order sensory/motor network (SMN) have better cognition and memory. We combined resting‐state and task‐based fMRI during an associative memory task to explore how this cross‐hierarchical contrast of rsFC is linked to memory aging.

**Method:**

We included resting‐state prior to (an object‐scene‐pair encoding) task fMRI from 16 young adults (YA; 24.6 ± 3.6 years; 7 females) and 42 cognitively normal older adults (OA; 76.8 ± 6.9 years; 24 females) in the Berkeley Aging Cohort Study. We subtracted the averaged rsFC within lower‐order regions from higher‐order ones, and correlated the cross‐hierarchy contrast of rsFC with age and memory task performance. Brain function contrast between the higher and lower order regions may also manifest as the temporal difference/delay of cortical activation, seen in propagating waves between hierarchical cortices. The propagating number (i.e., frequency) in each direction (“High‐to‐low” or “Low‐to‐high”) was therefore also correlated with memory performance and activation in anterior‐temporal (AT), posterior‐medial (PM), and hippocampal (HPC) memory regions during task‐fMRI.

**Result:**

Among OAs, cross‐hierarchy contrast of rsFC decreased with aging (Spearman’s ρ = ‐ 0.32, p = 0.040) and in females (p = 2.3 ×10^‐3^). OAs with lower rsFC contrast had worse memory during retrieval (ρ = 0.36, p = 0.019; Figure 1). Memory scores were not correlated with mean rsFC within higher‐order regions or lower‐order ones. Propagating number along the “Low‐to‐high” direction decreased in OAs. More propagation was associated with better memory and lower task‐evoked activation in AT, PM, and HPC regions (all ρ strength > 0.35, p < 0.030; Figure 2&3).

**Conclusion:**

Patterns of resting‐state functional connectivity related to hierarchical cortical organization are associated with aging and memory decline. In particular, slow propagating waves of cortical activation transiting across these hierarchies may serve a preparatory function, readying the brain for memory formation, a process that is affected by aging.